# Absolute Single Cavity Length Interrogation of Fiber-Optic Compound Fabry–Perot Pressure Sensors Through a White Light Non-Scanning Correlation Method

**DOI:** 10.3390/s19071628

**Published:** 2019-04-05

**Authors:** Zilong Guo, Wentao Lv, Wei Wang, Qingqing Chen, Xiongxing Zhang, Haibin Chen, Zhibo Ma

**Affiliations:** 1 School of Optoelectronic Engineering, Xi’an Technological University, Xi’an 710021, China; guozl@xatu.edu.cn (Z.G.); WenTaoLv1875647@163.com (W.L.); chenqing9301@163.com (Q.C.); zhangxiongxing@xatu.edu.cn (X.Z.); chenhaibin@xatu.edu.cn (H.C.); 2 Shaanxi Province Key Lab of Photoelectric Measurement and Instrument Technology, Xi’an Technological University, Xi’an 710021, China; 3 Key Lab of Micro/Nano Systems for Aerospace, Ministry of Education, Xi’an 710072, China; zbma@nwpu.edu.cn; 4 Shaanxi Key Lab of MEMS/NEMS, Northwestern Polytechnical University, Xi’an 710072, China

**Keywords:** fiber optic sensor, Fabry–Perot cavity, compound cavity, white light interferometry, non-scanning correlation method

## Abstract

A white light non-scanning correlation interrogation system was proposed and built to interrogate absolute length of the air cavity of fiber-optic compound Fabry–Perot pressure sensors for the extraction of pressure value. By carefully choosing thickness range and tilt angle of the optical wedge used for cavity length matching, correlation interferometric signal of the basal cavity can be naturally filtered out. Based on peak positioning by Fourier transform, bandpass filtering in frequency domain, inverse Fourier transform back to time domain, envelope fitting and zero fringe finding through a gravity center method, cavity length can be determined with an accuracy of 0.04%. The system was used for the interrogation of a fiber-optic compound Fabry–Perot pressure sensor under different pressures. For a pressure range of 0.1~2.9 Mpa, the linear relationship between the air cavity length and the gas pressure imposed was successfully extracted.

## 1. Introduction

Fiber-optic Fabry–Perot (FP) sensors, can be widely used for the accurate measurement of various quantities, such as temperature, strain/stress, refractive index, vibration, displacement, velocity, rotation, electric/magnetic field, and pH value. Furthermore, for their advantages of high accuracy, simple structure, tiny size, light weight, chemical passivity and anti-electromagnetic interference fiber-optic FP sensors have found many applications in petroleum, aviation, aerospace, and medical [1,2,3,4].

For the real applications of fiber-optic FP sensors, one of the key issues is how to get the measured quantity through cavity length interrogation. Nowadays, the most commonly used methods are classified into two categories, laser interference method [5,6,7], and white light interference method [8,9,10,11,12,13,14,15,16]. The laser interference method uses the interfering effect of a single continuous-wave laser to measure cavity length changes of an FP sensor, which is relatively simple, but only relative cavity length changes in a limited range can be measured, and the laser wavelength has to be stabilized at the Q-point of the fiber FP cavity. Losses in optical paths and environmental disturbances can result in serious influences on the system stability and measurement accuracy.

White light interference method is the most commonly used interrogation mechanism for absolute cavity length resolving in fiber-optic FP sensing applications, which includes spectral interrogation method and correlation method.

In white light spectral interrogation, usually, a wideband white light source or a wavelength tunable laser is used as the illumination source, an optical spectrum analyzer (OSA) is used as the detector, and a data processing algorithm based on Fourier transform [8,9] or digital correlation method [10,11] is used to extract the absolute cavity length. But the calculation is very complex and time-consuming and because of that the method cannot be used in conditions which require high-speed data acquisition rate. What is more, the resolution is limited by the bandwidth of the light source and the wavelength resolution of the OSA. In addition, a high-resolution OSA is very expensive, which makes the cost of the whole sensing system unacceptably high.

For the white light correlation method, with a broadband light source illuminating the fiber-optic FP sensor, a reference FP cavity with a tunable cavity length is used to generate a correlation interferometric signal between the two FP cavities, from which the cavity length of the fiber-optic FP sensor can be obtained. This is called scanning correlation interrogation [12]. Since the cavity length tuning of the reference FP cavity commonly is a mechanical process, the interrogation rate usually is relatively low. Fortunately, an air or a birefringent optical wedge (Fizeau interferometer) combined with a CCD line array can be used to directly obtain the correlation interferometric signal without temporal scanning, which can be called non-scanning correlation interrogation [13,14,15,16,17]. With the using of high-speed CCD or CMOS line array, the method has the potential to achieve high interrogation rate.

Most fiber-optic FP sensors simply have only one FP cavity, therefore, the cavity lengths are relatively easy to be resolved. But in special applications, like high temperature pressure sensing for harsh environment, limited by materials and fabrication processes, the fiber-optic FP sensors commonly contain two FP cavities, which are called fiber-optic compound FP pressure sensors. For example, external Fabry–Perot interfering (EFPI) sapphire or SiC pressure sensors [18,19,20] used in extremely high temperature conditions, commonly are composed of a relatively thick basal cavity formed by a substrate plate, and a relatively thin air cavity formed by front surface of the substrate plate and back surface of a pressure sensitive film. To achieve accurate pressure sensing, length or length change of the air cavity should be extracted precisely. The existence of the basal cavity will cause some troubles in the interrogation, which makes exact length acquisition of the pressure-sensitive air cavity difficult.

Till now, most papers on absolute cavity interrogation of fiber-optic compound FP pressure sensors are based on white light spectral interrogation [21,22], which has the potential or ability to interrogate two cavities at the same time, but usually with a low data acquisition rate. What is more, for the limitation comes from the spectral width of the wideband source and the disturbance of the basal cavity, interrogation resolution of the cavity length is relatively low. If the white light non-scanning correlation method is used, the correlation interferometric signal of the relatively thick basal FP cavity out of the optical thickness range of the optical wedge will be automatically filtered out. Therefore, by rational design, only the useful correlation interferometric signal of the pressure-sensitive air cavity will be left for the extraction of its cavity length. The white light non-scanning correlation method is very suitable for single cavity length interrogation of fiber-optic compound fiber FP pressure sensors.

The paper proposed a method to extract the absolute length of the pressure-sensitive air cavity of the fiber-optic compound FP pressure sensor by white light non-scanning correlation. Based on a peak positioning algorithm combined with Fourier transform, bandpass filtering in frequency domain, inverse Fourier transform, envelope fitting, and gravity center method, the absolute cavity length of the pressure-sensitive air cavity can be extracted. In addition, an experiment on the interrogation of a compound fiber-optic FP pressure sensor under different pressures was carried out to verify the feasibility and performance of the white light non-scanning correlation method for cavity length interrogation of compound fiber-optic FP pressure sensors.

## 2. System and Principle

### 2.1. Structure and Mechanism of the Fiber-Optic Compound FP Pressure Sensor

**Figure 1:**
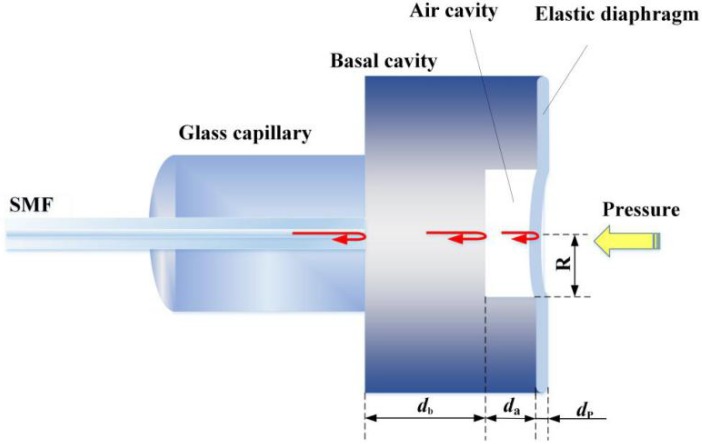
Schematic diagram of a typical EPFI sapphire pressure sensor.

The fiber-optic FP pressure sensor to be interrogated is an EFPI sapphire pressure sensor, as shown in Figure 1, which is composed of a basal cavity formed by a single sapphire crystal with a thickness of $d_{b}$, an air (or vacuum) cavity with a thickness of $d_{a}$, and a sapphire film in the front of the air cavity as the pressure sensitive elastic diaphragm. A pig-tailed optical fiber is attached to the back surface of the basal cavity through a glass capillary to guide light to illuminate the sensor and collect the reflected light. The front pressure sensitive film can be viewed as a flat circular diaphragm clamped at its circumference. When a uniform pressure is imposed, the elastic deformation of the film will result in a thickness change of the air cavity. The thickness variation of the air cavity $\Delta d_{a}$ in the axial center is in a linear relationship with the change of the pressure imposed, which can be expressed as [23,24]

(1) $$\Delta d_{a}=\frac{3R^{4}(1-\nu^{2})\Delta p}{16Ed_{P}^{3}},$$

where, ν is Poisson ratio, Δp is the change of the pressure imposed, E is the elastic modulus, R and $d_{P}$ are the radius and thickness of the pressure sensitive film, respectively. Through the linear relationship, pressure can be measured by length interrogation of the air cavity. The maximal pressure that can be measured is limited by the deflection limit of the elastic diaphragm. By the optimization of geometrical parameters—the film radius R and the film thickness $d_{P}$, the pressure measurement range and sensitivity can be determined purposely. Especially, by using (micro-electromechanical system) MEMS technology with materials, such as sapphire or SiC, high-pressure EFPI sensors can be accordingly designed and mass-fabricated.

### 2.2. White Light Non-Scanning Correlation Interrogation System and Principle

**Figure 2:**
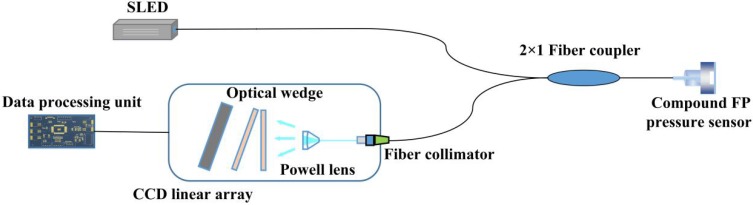
Schematic diagram of a white light non-scanning correlation interrogation system.

A white light non-scanning correlation interrogation system for single cavity (i.e., the air cavity) length interrogation of fiber-optic compound FP pressure sensors was built, as shown in Figure 2. The system is composed of a superluminescent light-emitting diode (SLED), a 2 × 2 fiber coupler, a fiber collimator, a Powell lens, an optical wedge, a CCD linear array, and a data processing unit.

The wideband light emitted by the SLED is coupled into the fiber-optic compound FP pressure sensor through the 2 × 2 fiber coupler, for the reflections at multiple surfaces of the sensor, after interferences in the basal and air cavities, part of the light is reflected back into the 2 × 2 fiber coupler. Half of the reflected light is coupled into the other fiber arm of the coupler. The light is coupled into free space as a collimated beam by a tiny fiber collimator. Then, the spatial light distribution is changed into a line-type profile by the beam-shaping of the Powell lens. The light carrying interferometric information of the fiber-optic compound FP pressure sensor then passes through the optical wedge. For their spatial correlation, a correlation interferometric signal is formed and received by a CCD linear array at the back surface of the optical wedge. Then the correlation interferometric signal is changed into an electric form. By the data processing unit, the position of the maximal intensity of the correlation interferometric signal is finally determined to get the absolute air-cavity length of the fiber compound FP pressure sensor. Since only the air cavity length is related to the pressure change, to filter out the interferometric signal from the basal cavity, and achieve a better resolution, the optical wedge should be optimally designed to cover the length of the air cavity only.

Light intensity distribution received by the CCD linear array after the optical wedge is determined by both the fiber FP pressure sensor and the optical wedge.

For a SLED with a spectral density $I_{0}\left(\lambda \right)$ in wavelength domain, the reflected light by a single FP cavity can be expressed by

(2) $$I_{\mathrm{FPr}}\left(\lambda \right)=\frac{R_{1}+R_{2}-2\sqrt{R_{1}R_{2}}\cos\frac{4\pi nd}{\lambda }}{1+R_{1}R_{2}-2\sqrt{R_{1}R_{2}}\cos\frac{4\pi nd}{\lambda }}I_{0}\left(\lambda \right),$$

in which, $R_{1}$ and $R_{2}$ are reflection ratios of the two interfaces of the FP cavity, n is the refractive index of the material filled in the FP cavity, and d is the cavity length.

For a low fineness FP cavity, Equation (2) can be rewritten by

(3) $$I_{\mathrm{FPr}}\left(\lambda \right)=\left(A+B\cos\frac{4\pi nd}{\lambda }\right)I_{0}\left(\lambda \right),$$

where, $A=R_{1}+R_{2}$ $B=-2\sqrt{R_{1}R_{2}}$.

For a low fineness compound FP pressure sensor, the reflected light can be expressed by

(4) $$I_{\mathrm{CFPr}}\left(\lambda \right)=\left(A_{a}+B_{a}\cos\frac{4\pi d_{a}}{\lambda }\right)\left(A_{b}+B_{b}\cos\frac{4\pi n_{b}d_{b}}{\lambda }\right)I_{0}\left(\lambda \right),$$

where, $A_{a}$, $B_{a}$, $A_{b}$, $B_{b}$ are four reflection constants for the air and basal cavities, $d_{a}$ and $d_{b}$ are lengths of the two FP cavities of the compound FP pressure sensor, respectively, $n_{b}$ is the refractive index of the basal cavity.

When the light passes through an air-gap optical wedge with a tilt angle of θ, at a position of x, the transmitted light can be expressed by

(5) $$I_{\mathrm{Wt}}\left(x\right)=\frac{{\left(1-R_{3}\right)}^{2}}{1+R_{3}^{2}-2R_{3}\cos\frac{4\pi l}{\lambda }}I_{\mathrm{CFPr}}\left(\lambda \right),$$

where, we suppose the two inner surfaces of the air-gap optical wedge have the same reflection ratio of $R_{3}$, and, l is the separation of the two inner surfaces, which is written by

(6) $$l=l_{0}+x\tan\theta ,$$

in which, $l_{0}$ is an initial separation of the two inner surfaces at one end of the air-gap optical wedge. Considering light distributions in both spatial and wavelength domains, the mathematical model of the light obtained by the CCD linear array at a position of x can be expressed as

(7) $$I_{OUT}\left(x\right)=\eta \left(x\right)\int_{\lambda_{\min}}^{\lambda_{\max}}\left(A_{a}+B_{a}\cos\frac{4\pi d_{a}}{\lambda }\right)\left(A_{b}+B_{b}\cos\frac{4\pi n_{b}d_{b}}{\lambda }\right)I_{0}\left(\lambda \right)\frac{{\left(1-R_{3}\right)}^{2}}{1+R_{3}^{2}-2R_{3}\cos\frac{4\pi l}{\lambda }}I_{0}\left(\lambda \right)d\lambda ,$$

where, η(x) is the intensity spatial distribution introduced by the fiber collimator and Powell lens, $\lambda_{\min}$~$\lambda_{\max}$ is the wavelength range of the SLED source.

From the mathematical point of view, Equation (7) can be regarded as a correlation function between the cavity lengths of the compound FP pressure sensor and the inner separation l of the air-gap optical wedge. $I_{OUT}\left(x\right)$ can be called correlation interferometric signal.

The air-gap optical wedge is the key device for the correlation operation, it is used to achieve a spatial scanning of the correlation operation with the optical thickness of each FP cavity of the compound fiber sensor. For the mixing effect of Equation (4), there are four optical lengths $d_{a}$, $n_{b}d_{b}$, $n_{b}d_{b}-d_{a}$, and $n_{b}d_{b}+d_{a}$ to do correlation with l. When the inner separation $l=l_{0}+x\tan\theta$ of the optical wedge at a position is equal to one of the four optical lengths $d_{a}$, $n_{b}d_{b}$, $n_{b}d_{b}-d_{a}$, or $n_{b}d_{b}+d_{a}$, the correlation interferometric signal will reach a light intensity maximum at the position x.

For a SLED source with a center wavelength of $\lambda_{0}$ and a 3 dB spectral width of Δλ in wavelength domain, then it will have a coherence length of $L_{c}=\lambda_{0}^{2}/\Delta \lambda$, to distinguish each correlation interferometric signal without obvious overlapping to others, the optical length differences between $d_{a}$, $n_{b}d_{b}$, $n_{b}d_{b}-d_{a}$, or $n_{b}d_{b}+d_{a}$ should be larger than half the coherence length $L_{c}$.

For a real fiber-optic compound FP pressure sensor, usually, $d_{b}>>d_{a}$. To achieve pressure sensing through accurate interrogation of $d_{a}$, an air-gap optical wedge that only covers the variation range of the short air-cavity length $d_{a}$ can be designed. For the reason that $n_{b}d_{b}$, $n_{b}d_{b}-d_{a}$, or $n_{b}d_{b}+d_{a}$ are far out of the range of l, the correlation interferometric signals of the three quantities will be averaged as a flat DC signal. Only the length $d_{a}$ of the pressure-sensitive air cavity will form a correlation interferometric maximum in the output. Thus, through the peak positioning of the correlation interferometric signal, pressure-sensitive air cavity length of the fiber-optic compound FP pressure sensor can be obtained by the position x and the wedge angle θ.

For a fiber-optic compound FP pressure sensor, in the white light non-scanning correlation interrogation system, through the rational design of the maximal and minimal thickness of the optical wedge, the interferometric signal of the basal cavity with no relationship with the pressure to be measured can be effectively filtered out.

### 2.3. Peak Positioning Algorithm of the Correlation Interferometric Signal

**Figure 3:**
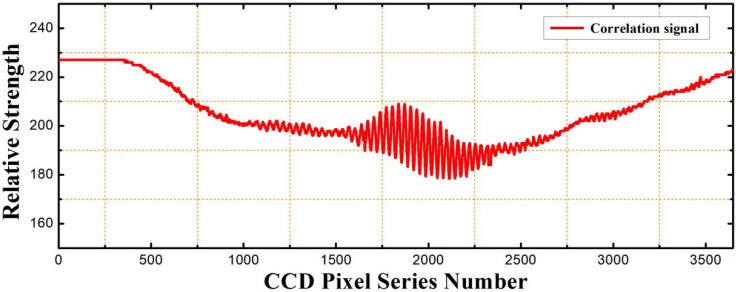
Typical correlation interferometric signal for a fiber-optic compound FP pressure sensor with a basal cavity length of 650 μm, and an initial air-cavity length of 81.873 μm under one standard atmosphere pressure. The air-gap optical wedge used is with a thickness range of 60~95 μm.

A typical correlation interferometric signal for a fiber-optic compound FP sensor with a basal cavity length of 650 μm, and an initial air-cavity length of 81.873 μm under one standard atmosphere pressure is shown in Figure 3. The air-gap optical wedge used was with a thickness range of 60~95 μm. It can be found that no obvious signal from the basal cavity was shown in the measuring range. So, the optical wedge can be viewed as a natural filter for the basal cavity. For the correlation interferometric signal, it has the following characteristics: Firstly, it has a background signal, which is related to various parameters of the fiber collimator, Powell lens, and the distance between the Powell lens and the CCD linear array. The background signal can be viewed as a relative light intensity distribution on the CCD linear array generated from the reflected light of a uniform reflection surface. In addition, there are high-frequency noises that come from the sampling circuit.

To accurately get the air-cavity length, the main task is to precisely find the peak position of the correlation interferometric signal. However, as the serious disturbances come from the low-frequency background signal and the high-frequency noises, the peak position of the correlation interferometric signal cannot be accurately determined directly through peak searching, which results in a low resolution of cavity length extraction. To solve the problem, we propose an algorithm procedure composed of Fourier transform, bandpass filtering in the frequency domain, inverse Fourier transform back to time domain, envelope fitting and zero fringe finding through a gravity center method. Details of the algorithm are as follows:

Firstly, the correlation interferometric signal is Fourier transformed into the frequency domain, then, a bandpass filter is used to filter the low-frequency background signal and high-frequency noises.

After filtering, the signal is reversely transformed back to the time domain again. The background signal and high-frequency noises can be effectively filtered out if a proper bandpass filter is selected.

Then the envelope of the filtered correlation interferometric signal is fitted through a method of instantaneous effective value extraction. The effective value of a signal is also called root mean square value, which can be used to describe the signal intensity. The instantaneous effective value of the filtered correlation interferometric signal is an effective value in a short time duration, which can be calculated by

(8) $$V=\sqrt{\frac{2}{m}\sum_{i=1}^{m}{\left(I_{OUT}(x_{i})-\bar{I}\right)}^{2}},$$

where, V is the instantaneous effective value of the signal, m is the signal width of the instantaneous effective value, i.e., pixel number of the filtered correlation interferometric signal in a signal period, $I_{OUT}(x_{i})$ is the relative intensity of the signal on i-th pixel in the selected effective signal width, and $\bar{I}$ is the average of the filtered correlation interferometric signal.

After getting the envelope through instantaneous effective value extraction, firstly, the peak position of the envelope is found by direct numerical value comparison, then, choose an effective signal range with the peak position as the center. Using the method of gravity center in the signal range to find the gravity center as the real peak of the correlation signal. The calculation of the gravity center follows the equation,

(9) $$G_{x}=\frac{\sum_{j=a}^{b}x_{j}{I^{\prime }}_{OUT}(x_{j})}{\sum_{j=a}^{b}x_{j}},$$

where, $G_{x}$ is the position of the gravity center, a and b are the start and end of the effective signal range, respectively, $x_{j}$ and ${I^{\prime }}_{OUT}(x_{j})$ are the j-th x coordinate and the j-th relative intensity of the filtered correlation interferometric signal, respectively.

**Figure 4:**
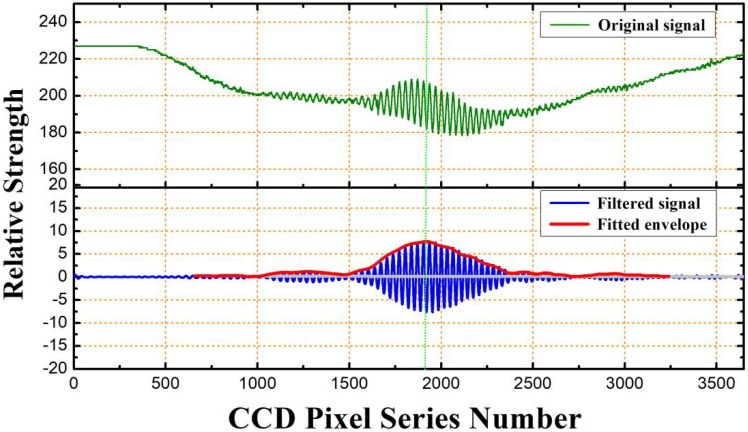
Peak positioning of the correlation interferometric signal.

As an example, in Figure 4, the peak positioning process for a correlation interferometric signal is shown. An original correlation signal, with background signal and high-frequency noises, is transformed into the frequency domain through Fourier transform, then, after the filtering of a bandpass filter, the signal is transformed back, it can be seen that background signal and high-frequency noises have been filtered out, the envelope is found through instantaneous effective value extraction, with the envelope center to find an effective signal range, the gravity center method is then used to finally precisely determine the center of the correlation signal. For a cavity length of 81,873 nm, the peak position was found at pixel 1923.

## 3. Experimental Verification

### 3.1. Experimental Setup

**Figure 5:**
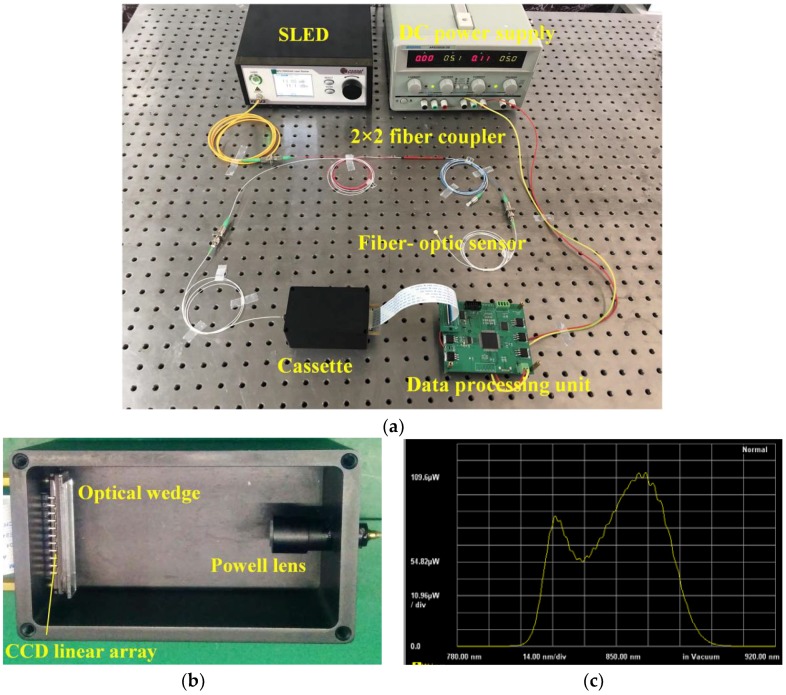
Experimental setup of the white light non-scanning correlation interrogation system. (**a**) Picture of the whole system, (**b**) inner structure of the cassette, (**c**) optical spectrum of the SLED.

According to the schematic diagram of Figure 2, a white light non-scanning correlation interrogation system was built. Its picture is shown in Figure 5a. A bench-top SLED with a center wavelength of 850 nm, and 3 dB spectral bandwidth of 58 nm was used as the wideband light source, of which, the output optical spectrum is shown in Figure 5c. It can be calculated that the SLED has a coherence length of 12.5 μm. An optical isolator was internally integrated into the SLED. The 2 × 2 fiber coupler (Thorlabs, TW850R5A2) was a wide band type with a center wavelength of 850 nm and a minimum transmission range of ±100 nm. For the coupling and beam-shaping of the light beam output from the optical fiber, a tiny fiber collimator and a Powell lens with a fan angle of 60° were used. At a distance of 50 mm, the intensity distribution of the light was reshaped into a line. A CCD linear array was placed at the place, which is a 3648 linear array (fabricated by Toshiba Semiconductor, TCD1304DG), the size of each pixel is 8 μm × 200 μm, the total photosensitive length is 29.2 mm, the dynamic range is 300, and the minimal integration time can reach a value of 10 μs under an electronic shutter mode. It should be noted that the output current signal of the CCD is inversely proportional to the light intensity.

In front of the CCD linear array, an optical wedge fabricated by two optical glass plates was placed. The designed thickness range is from 50 to 100 μm. Two narrow glass plates with a thickness of 50 μm and 100 μm, respectively, were used to pad at the two ends of the optical glass plates, respectively, and then an UV-cured adhesive was used to fix all the glass plates. However, for the thickness of the adhesive and its flowability, the final measurement range was found to be between 60 and 95 μm.

To avoid adverse effects of background light on the CCD linear array, the fiber collimator, the Powell lens, the optical wedge, and the CCD linear array are all assembled and sealed in a metal cassette, as seen in Figure 5b. Furthermore, the cassette can also protect the optical surfaces of the devices from the contamination of dust in air. For the using of Powell lens to achieve a line beam profile, the whole optical system was very compact.

Finally, a self-designed data processing unit with FPGA as the central processing chip was used to drive the CCD linear array and process the obtained data. The peak positioning algorithm of the correlation interferometric signal and the cavity length calculating program were written through a Verilog HDL language in the FPGA chip.

### 3.2. System Calibration

After finding out the peak position (or the pixel number of the CCD linear array) of the correlation interferometric signal, absolute cavity length of any fiber-optic FP sensor can be calculated through Equation (6). However, the relationship between the thickness of the optical wedge and the pixel number cannot be known without calibration. The reasons are as follows. In the assembling of the optical wedge and the CCD linear array, it is hard to achieve the minimal thickness of the optical wedge precisely at the first pixel of the CCD linear array. Furthermore, for the fabrication process of the optical wedge, we cannot guarantee the minimal or maximal thickness of the optical wedge exactly equal the designed values (In this paper, the designed minimal and maximal thickness of the optical wedge is 50 μm and 100 μm, respectively, and the real measurement range was between 60 and 95 μm). Therefore, the corresponding relationship between the thickness of the optical wedge and the CCD pixel serial number should be precisely calibrated before the white light non-scanning correlation interrogation system can be used.

**Figure 6:**
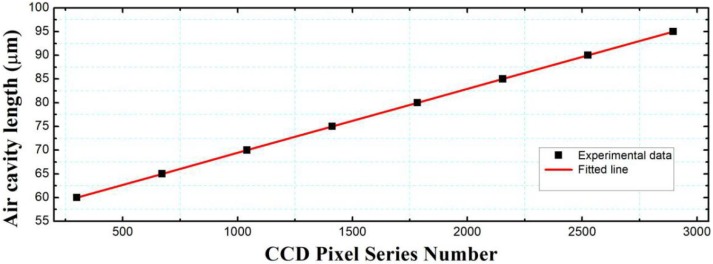
Calibration of the relationship between the wedge thickness and the CCD pixel serial number.

In the calibration experiment, eight single fiber FP air cavities with cavity lengths of 60.003 μm, 65.002 μm, 69.998 μm, 74.999 μm, 80.001 μm, 84.998 μm, 90.002 μm, and 94.999 μm, respectively, are used to achieve the calibration of the system through a linear fitting. By finding the CCD pixel serial number of the correlation interferometric signal’s peak of each fiber FP cavity, and linearly curve fitting the experimental data of the eight points, the relationship between the cavity length versus the CCD pixel serial number was obtained, as shown in Figure 6. We have l=0.0134779n+55.95685, where n is the CCD pixel serial number, l is the inner separation of the air-gap optical wedge (μm). The degree of fitting is $R^{2}=0.9999$, the accuracy in the full range of 60~95 μm reached a value of 0.04%.

### 3.3. Pressure Measurement Based on the Interrogation System

**Figure 7:**
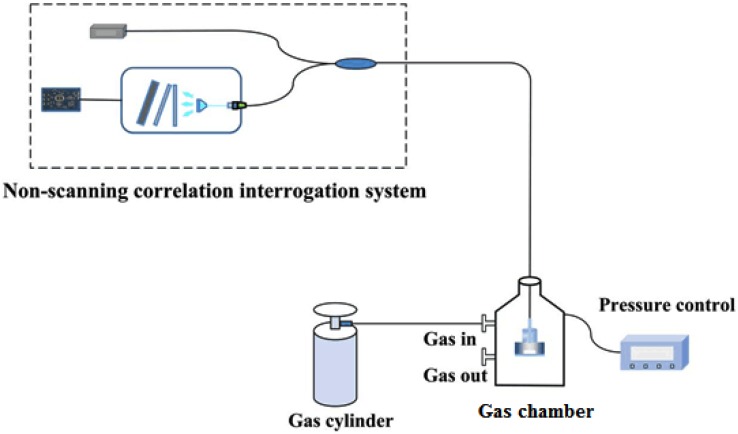
Cavity length interrogation of a compound fiber FP pressure sensor under a pressure testing experiment.

To verify the feasibility of the white light non-scanning correlation interrogation system for single cavity (i.e., the air cavity) length demodulation of fiber-optic compound FP pressure sensors, an EFPI sapphire pressure sensor was connected to the interrogation system and put into a gas chamber for a pressure test. For the fabrication of the EFPI sapphire pressure sensor, three layers of single crystal sapphires were used. One layer was used as the basal cavity. One layer was firstly drilled with a through hole with a certain radius, then it was connected with the first layer by direct bonding under high temperature. After thermal treatment, the layer with the through hole was thinned to the required thickness by mechanical polishing. The third layer was attached to the through-hole layer by the high-temperature direct bonding method again and also was thinned by mechanical polishing to form the pressure sensitive film. Because the EFPI sapphire pressure sensor was completely composed of single sapphire crystals, no other adhesives or materials were used in the pressure sensitive structure, it has good response to pressure imposed. The fiber-optic compound FP pressure sensor tested has a basal cavity length of 650 μm, an initial air cavity length of 81.873 μm, thickness and radius of the pressure sensitive film are 27 μm and 0.65 mm, respectively. Through theoretical simulation, it was shown that for a pressure change from 0 to 3.0 MPa, the elastic diaphragm will have a deformation from 0 to 12.1 μm. The pressure testing system was composed of a gas cylinder, a gas chamber, and a precise pressure controller, as seen in Figure 7.

A commercial gas pressure controller (Druck, PACE-5000, pressure range: 21.1 MPa) with an accuracy of ±0.003% FS was used for the pressure control and monitoring. The gas used for pressure testing was high purity nitrogen. The pressure in the gas chamber was controlled to increase from 0.1 MPa to 2.9 MPa with a step of 0.2 MPa. For each pressure, it was left for five minutes to achieve a static state of pressure equilibrium, at the same time, the self-built white light non-scanning correlation interrogation system was used to measure the absolute length of the pressure-sensitive air cavity. The experiment was carried out in room temperature.

**Figure 8:**
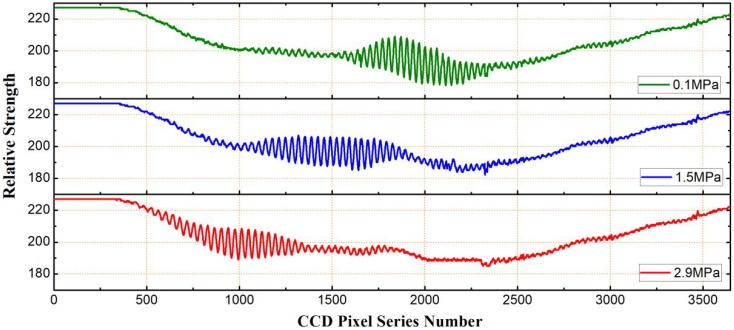
Correlation interferometric signals at three different pressures of 0.1 MPa, 1.5 MPa, and 2.9 MPa.

**Figure 9:**
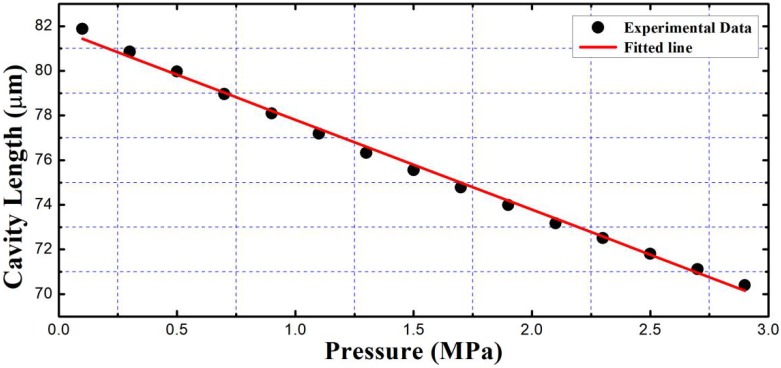
Relationship between air cavity length and pressure imposed.

In Figure 8, correlation interferometric signals for three different pressures of 0.1 MPa, 1.5 MPa, and 2.9 MPa were given. It was found that pixel serial numbers of the three peaks were 1923, 1487, and 1049, respectively. Correspondingly, the cavity lengths were 81.873 μm, 75.984 μm, and 70.096 μm, respectively. The peaks are at three different positions. It can be seen that, in the pressure increasing process, the correlation interferometric signal was changing its position continuously from left to right, i.e., a thickness decreasing direction of the optical wedge, that means, the air-cavity length of the EFPI sapphire pressure sensor was decreasing with the increase of the pressure imposed. The relation between the pressure and the cavity length is shown in Figure 9. It can be seen that the pressure and the cavity length is in a linear relationship, which was faithful to the theoretical expectation of Equation (1). Through linear fitting, we have $d_{a}=-4.02842P+81.83935$, where, P is the pressure imposed (Mpa), $d_{a}$ is the air-cavity length (μm). As pressure changed from 0.1 Mpa to 2.9 Mpa, the full cavity length changing calculated was 11.777 μm, the cavity length–pressure sensitivity was 0.42 nm/kPa, and the nonlinear error was about 3.78%.

From the pressure experiment, we can see that, the white light non-scanning correlation interrogation system can achieve accurate absolute air-cavity length interrogation of compound fiber FP pressure sensors. The white light non-scanning correlation interrogation system for fiber-optic compound Fabry–Perot FP sensors can be easily used for higher pressure measurement. To achieve the purpose, according to Equation (1), we only need to increase the thickness or reduce the radius of the pressure-sensitive film in the fiber-optic compound FP pressure sensor. At the same time, the interrogation system does not need any change.

## 4. Conclusions

A white light non-scanning correlation interrogation system was proposed to achieve single cavity length interrogation of the air cavity of fiber-optic compound FP pressure sensors for pressure extraction. Through the rational choice of the thickness range of the optical wedge, the interferometric signal of the basal cavity without relation with the imposed pressure can be effectively filtered. For fiber-optic compound FP pressure sensor with a basal cavity of 650 μm and an initial air cavity length of 81.873 μm under one standard atmosphere pressure, a correlation interrogation system with measurement range of 60~95 μm was built and calibrated. A peak positioning method combined by Fourier transform, bandpass filtering in the frequency domain, inverse Fourier transform back to the time domain, envelope fitting and zero fringe finding through a gravity center method was proposed and written in the FPGA chip of the data processing unit. The system was used for the real interrogation of the fiber-optic compound FP pressure sensor for a pressure range of 0.1~2.9 MPa, the relationship between the absolute cavity length of the air cavity and the pressure imposed was given, which was with a cavity length–pressure sensitivity of 0.42 nm/kPa, and a nonlinear error of 3.78%. The experiment shows that the white light non-scanning correlation interrogation system, with proper design of measurement range, can successfully achieve single cavity length interrogation of fiber-optic compound FP pressure sensors. Compared with those fiber-optic compound FP pressure sensors interrogated based on white light spectral method, absolute length of the pressure-sensitive air cavity is obtained more directly, no expensive OSA is needed. By using Powell lens to achieve beam-shaping, if the SLED is also replaced by a small OEM device or driven by a self-designed circuit, the whole interrogation system can be very compact, even a handheld system can be realized.
